# A Novel Function for *Arabidopsis* CYCLASE1 in Programmed Cell Death Revealed by Isobaric Tags for Relative and Absolute Quantitation (iTRAQ) Analysis of Extracellular Matrix Proteins*[Fn FN2]

**DOI:** 10.1074/mcp.M114.045054

**Published:** 2015-04-10

**Authors:** Sarah J. Smith, Johan T. M. Kroon, William J. Simon, Antoni R. Slabas, Stephen Chivasa

**Affiliations:** From the ‡School of Biological and Biomedical Sciences, Durham University, Durham DH1 3LE, United Kingdom

## Abstract

Programmed cell death is essential for plant development and stress adaptation. A detailed understanding of the signal transduction pathways that regulate plant programmed cell death requires identification of the underpinning protein networks. Here, we have used a protagonist and antagonist of programmed cell death triggered by fumonisin B1 as probes to identify key cell death regulatory proteins in *Arabidopsis*. Our hypothesis was that changes in the abundance of cell death-regulatory proteins induced by the protagonist should be blocked or attenuated by concurrent treatment with the antagonist. We focused on proteins present in the mobile phase of the extracellular matrix on the basis that they are important for cell–cell communications during growth and stress-adaptive responses. Salicylic acid, a plant hormone that promotes programmed cell death, and exogenous ATP, which can block fumonisin B1-induced cell death, were used to treat *Arabidopsis* cell suspension cultures prior to isobaric-tagged relative and absolute quantitation analysis of secreted proteins. A total of 33 proteins, whose response to salicylic acid was suppressed by ATP, were identified as putative cell death-regulatory proteins. Among these was CYCLASE1, which was selected for further analysis using reverse genetics. Plants in which *CYCLASE1* gene expression was knocked out by insertion of a transfer-DNA sequence manifested dramatically increased cell death when exposed to fumonisin B1 or a bacterial pathogen that triggers the defensive hypersensitive cell death. Although pathogen inoculation altered *CYCLASE1* gene expression, multiplication of bacterial pathogens was indistinguishable between wild type and *CYCLASE1* knockout plants. However, remarkably severe chlorosis symptoms developed on gene knockout plants in response to inoculation with either a virulent bacterial pathogen or a disabled mutant that is incapable of causing disease in wild type plants. These results show that CYCLASE1, which had no known function hitherto, is a negative regulator of cell death and regulates pathogen-induced symptom development in *Arabidopsis*.

Programmed cell death (pcd)^1^ is a genetically controlled dismantling of cells, which is indispensable for plant development and stress-adaptive responses. In development, pcd is invoked to facilitate xylem tracheary element differentiation, to remodel leaf shape, and to delete ephemeral cells and organs such as embryonic suspensor cells (1–3). In response to drought stress, pcd is used to break root apical meristem dominance in order to remodel root system architecture as an adaptive response to water deficit (4). Additionally, a specialized form of pcd known as the hypersensitive response kills plant cells at the epicenter of attack by certain pathogens, which activate the effector-triggered immune response (5, 6). A detailed understanding of the signal transduction pathways that trigger, propagate, and terminate plant pcd requires identification of the key components of the underlying protein networks. Our group has been using *Arabidopsis* cell death induced by fumonisin B1 (FB1) as an experimental system to study plant pcd and identify the key regulatory proteins (6).

FB1, a mycotoxin that triggers cell death in both animal and plant cells (8, 9), disrupts sphingolipid biosynthesis via inhibition of ceramide synthase (10). Several proteins directly involved in sphingolipid biosynthesis and metabolism have been shown to regulate FB1-induced plant pcd because of their influence on levels of metabolic intermediates, such as long chain bases (LCBs), which act as second messengers of plant cell death. For example, activity of serine palmitoyltransferase, the enzyme catalyzing the first rate-limiting step in sphingolipid biosynthesis, strongly controls *Arabidopsis* sensitivity to FB1 (11). Serine palmitoyltransferase has two subunits – LCB1 and LCB2. Resistance to FB1-induced death is manifested in *Arabidopsis* loss-of-function mutants of *LCB1* (12) and *LCB2a* (13) genes. Overexpression of endogenous *Arabidopsis* 56 amino acid polypeptides that interact with and stimulate serine palmitoyltransferase activity increases sensitivity to FB1, whereas RNA interference lines have reduced sensitivity to the mycotoxin (11).

Although exogenous ceramide can suppress FB1-induced death in animal cells (14), it fails to block cell death in *Arabidopsis* (15), indicating that other factors work in concert with ceramide depletion in pcd induction in *Arabidopsis*. Identification of these factors is essential to the understanding of general pcd regulation in plants, given that *Arabidopsis* responses to FB1 share common features with the pathogen-induced hypersensitive response (15). Clues that may lead to mechanistic details of pcd could arise from focusing on known regulatory signals that control FB1-mediated responses. FB1-induced cell death is regulated by extracellular ATP (eATP) (16) and the plant defense hormone, salicylic acid (SA) (17). NahG transgenic plants, which degrade SA, are resistant to FB1 as are *pad4–1* mutants, which have an impaired SA amplification mechanism (17). Mutants that constitutively accumulate greater amounts of SA, *cpr1* and *cpr6*, manifest increased susceptibility to FB1 (17). Thus, SA functions as a positive regulator of FB1-induced pcd. In contrast, eATP is a negative regulator of FB1-triggered pcd in *Arabidopsis*. Accordingly, FB1 activates eATP depletion prior to onset of death and addition of exogenous ATP to FB1-treated *Arabidopsis* cell suspension cultures blocks pcd (16). This suggests that SA- and eATP-mediated signaling converge onto the signal transduction cascade activated by FB1 to promote or inhibit pcd, respectively.

We have developed an experimental system, which harnesses the effects of exogenous ATP and SA on FB1-induced death, to identify important proteins that regulate *Arabidopsis* pcd. It utilizes *Arabidopsis* cell suspension cultures treated with these compounds and proteomic analyses restricted to the mobile phase of the extracellular matrix. The extracellular matrix proteome consists of cell surface proteins fully or partially embedded in the plasma membrane, proteins immobilized in the cell wall, and soluble mobile proteins in the apoplastic fluid – the mobile phase. The rationale for this is predicated on the hypothesis that cells constantly communicate with their neighbors by releasing and sensing signal molecules in the mobile phase (18). *Arabidopsis* has more than 600 plasma membrane receptor kinases (19) and ∼400 G-protein-coupled receptors (20, 21), which sense extracellular signals at the cell surface and activate a cytoplasmic response. We hypothesize that upon receiving an exogenous chemical, cell–cell signaling is activated either by directly binding the chemical if it has a cell surface receptor, or by modulating signal regulatory proteins in the mobile phase to reset the communication and transmit new signals. Therefore, in this study, we used ATP and SA treatments to identify pcd regulatory proteins in the mobile phase of the *Arabidopsis* extracellular matrix. We provide a novel extracellular matrix putative cell death regulatory protein network and present evidence validating the role of CYCLASE1 in FB1- and pathogen-induced pcd and the control of disease symptoms.

## EXPERIMENTAL PROCEDURES

### 

#### 

##### Plant Material, Growth Conditions, and Treatments

The T-DNA insertion mutant line of *Arabidopsis* from the JIC SM collection (GT_5_42439) (22) was obtained from the Nottingham *Arabidopsis* Seed Stock Centre (Nottingham, UK). An *Arabidopsis* transposon-tagged line (RATM13_3839_1) (23, 24), developed by the plant genome project of RIKEN Genomic Sciences Centre, was ordered from RIKEN (Tsukuba, Japan). Plants were grown at 23 °C with a 16 h photoperiod at ∼150 μmolm^−2^sec^−1^ under cool white fluorescent lights. Cell suspension cultures of *Arabidopsis thaliana* derived from tissue of ecotype Landsberg *erecta* were maintained as described previously (25). All chemicals and growth media were purchased from Sigma (http://www.sigmaaldrich.com). Stock solutions of ATP and salicylic acid were prepared fresh in water and adjusted to pH 6.7 prior to application. FB1 stock solutions were prepared in 70% methanol. Cell cultures were treated by adding the appropriate volume of chemical into the growth medium, whereas leaves were infiltrated with the solutions into the apoplast using a syringe without a needle. All plants were used for experiments 4–5 weeks after sowing, whereas 30 ml cell cultures at 3 days post-subculturing were adjusted to a density of 5% (w/v) and treated by addition of appropriate solutions into the growth medium.

##### RNA Analysis

RNeasy Plant Kit (Qiagen, Crawley, UK) with on-column DNase treatment was used to extract total RNA from *Arabidopsis* leaf tissues according to the manufacturer's instructions. A previously described protocol (26) was used for first strand cDNA synthesis using 2 μg RNA template, oligo-(dT)_15_ (Promega, Southampton, UK), and SuperScript III reverse transcriptase (Invitrogen, Paisley, UK). For PCR reactions, the following primer pairs were used: *CYCLASE1* (At4g34180) 5′-AACATCCAACACCGACAAGCGGC-3′ and 5′- AACATCCAACACCGACAAGCGGC-3′; *ACTIN2* (At3g18780) 5′-GGATCGGTGGTTCCATTCTTG-3′; and 5′-AGAGTTGTCACACACAAGTG-3′.

##### Pathogen Infection Assays

*Pseudomonas syringae* pv. *tomato* strain DC3000, and the derivative DC3000-hrcC and DC3000-avrRpm1 strains, were grown overnight at 28 °C on King's B agar supplemented with rifampicin. Colonies from agar plates were resuspended in water to an inoculum density of 10^6^ colony forming units/ml. Three leaves per plant were syringe-infiltrated with the inoculum. Triplicate plants of each genotype were inoculated in this way and the bacterial titer in the leaf tissues assayed 3 days postinoculation for DC3000 and DC3000-avrRpm1, or 6 days post-inoculation for DC3000-hrcC. To determine bacterial titer, a pooled sample of 3 mm-diameter leaf discs, one from each of three replicate plants, was homogenized in sterile water and 10-fold dilutions of the homogenate plated out on agar plates with rifampicin. The number of colony forming units per square centimeter of infected leaf was calculated, converted to log scale, and the averages analyzed by Student's *t* test.

##### Cell Death Assays

Leaf discs of 8 mm diameter were cored from 4-week-old *Arabidopsis* plants and floated on 9 ml of 5 μm FB1 solution in a Petri-dish. A total of five-replicate Petri-dishes per genotype were generated, with each dish containing eight leaf discs originating from 10 different plants. The dishes were incubated for 48 h in the dark and transferred to a 16-hour photoperiod thereafter. Cell death progression was monitored by measuring conductivity of the FB1 solution in each dish every 24 h from 48 h onwards. To monitor pathogen-induced cell death, leaves were infiltrated with ∼10^7^ colony forming units/ml *Pseudomonas syringae* pv. *tomato* strain DC3000-avrRpm1. Discs of 8 mm diameter were immediately cored from the inoculated leaves and floated on 9 ml deionized water in a Petri-dish. Five-replicate dishes per genotype were generated, with each dish containing 10 discs from 10 different plants. Conductivity of the deionized water was measured every hour until 4 h and every 2 h from then until 12 h.

##### Cell Culture Treatments and Protein Extraction

Cell cultures were treated with 200 μm SA or a combination of 200 μm SA + 200 μm ATP. Controls were treated with an equivalent volume of sterile water. After 48 h, the cells were separated from the growth medium by filtration using a Mira cloth. Proteins secreted into the growth medium were recovered by precipitation in 80% acetone at −20 °C. The precipitates were resolubilized in a solution containing 9 m urea/2 M thiourea/4% (w/v) CHAPS. Each of the treatments and the control had three biological replicates, giving rise to a total of nine samples. Differential protein expression was analyzed using isobaric tags for relative and absolute quantitation (iTRAQ), and 2D-DiGE was used as a tool to confirm quantitative data from iTRAQ on a few selected proteins.

##### Sample Labeling and iTRAQ Analysis

Protein samples were acetone-precipitated and resuspended in 50 mm triethylammonium bicarbonate buffer containing 0.1% SDS. Prior to digestion, 75 μg of each protein sample were sequentially reduced and alkylated with tris(2-carboxyethylphosphine) (TCEP) and methyl-methane-thiol-sulfonate (MMTS), respectively. Protein digestion was at a 1:10 trypsin ration. The digested peptides were vacuum-dried, resuspended in triethylammonium bicarbonate buffer (pH 8.5), and labeled with 4-plex iTRAQ reagent kits (Applied Biosystems, Foster City, USA) for 1 h at room temperature as previously described (27). Control, SA, and ATP+SA samples were labeled with the 114, 115, and 116 iTRAQ tags, respectively. The three samples of each individual replicate experiment were pooled, vacuum-dried, and processed separately from the other replicates.

The pooled sample was resuspended in 3 ml of buffer A (10 mm K_2_HPO_4_/25% acetonitrile, pH 2.8) and separated on the Poly-LC strong cation exchange column (200 × 2.1 mm) at 200 μl/min on an Ettan LC (GE Healthcare) HPLC system. Peptide separation was performed using a biphasic gradient of: 0–150 mm KCl over 11.25 column volumes and 150–500 mm KCl in buffer A over 3.25 column volumes. A total of 52 × 200 μl fractions were collected over the gradient, but some were pooled to give a final total of 30 fractions that were dried down and resuspended in 90 μl of 2% acetonitrile/0.1% formic acid. Aliquots of 20 μl from each fraction were analyzed by LC-MS/MS using a nano-flow Ettan MDLC system (GE Healthcare) attached to a hybrid quadrapole-TOF mass spectrometer (QStar Pulsar *i*, Applied Biosystems, Foster City) coupled to a nanospray source (Protana) and a PicoTip silica emitter (New Objective, Woburn, MA). Samples were loaded and washed on a Zorbax 300SB-C18, 5 mm, 5 × 0.3 mm trap column (Agilent, Stockport, UK) and online chromatographic separation performed over 2 h on a Zorbax 300SB-C18 capillary column (3.5 × 75 μm) with a linear gradient of 0–40% acetonitrile, 0.1% formic acid at a flow rate of 200 nl/minute. Applied Biosystems Analyst software version 1.1 was used acquire all MS and MS/MS data switching between the survey scan (1 × 1 s MS) and three product ion scans (3 × 3 s MS/MS) every 10 s. Ions in the range of 2^+^ to 4^+^ charge state and with TIC > 10 counts selected for fragmentation.

##### Mass Spectra Data Analysis

Protein Pilot software version 2.0.1 (Applied Biosystems) was used to process all MS/MS data files using the *Arabidopsis* TIGR.fas database containing 27,855 protein sequences (downloaded in August 2007). MS and MS/MS tolerances were set to 0.15 and 0.1Da, respectively, and analysis and search parameters were set as: iTRAQ 4-plex labeling, trypsin digestion with allowance for a single missed cleavage, and only two amino acid modifications viz. MMTS-alkylated cysteine and oxidized methionine. Quantitative data were obtained from the iTRAQ tags within the mass spectra. In order to reduce protein redundancy and determine protein identification confidence scores (ProtScores) from the ProID output for each fraction, the data from all fractions were combined, analyzed, and reported using ProGroup software (Applied Biosystems, Foster City, USA). A protein identification threshold of 1.3, which retains only proteins identified with a 95% confidence, was applied to the data sets. Systematic errors arising from possible unequal mixing of labeled peptides were excluded by applying a bias correction factor. Its calculation is based on the assumption that most proteins do not change, so the software identifies the median average protein ratio and corrects it to unity, and then applies this factor to all quantitation results. In order to measure the false discovery rate the peptide mass spectra data sets were used to search a decoy peptide database, created by reversing peptide sequences of all entries in the database used in this study. Aggregate false discovery rate (FDR) was calculated as: FDR (%) = 100 × (2 × Decoy IDs)/Total IDs. Decoy IDs is the number of “proteins” identified from the decoy database that pass the thresholds set for identifying proteins in the real database. Total IDs is the number of proteins identified from the real database using the same peptide data set.

The data sets were manually filtered sequentially to exclude peptides leading to ≥2 protein identifications. From the filtered data sets, only those proteins identified across all three replicate experiments were retained for further analysis. A further filter applied to the data was to include only proteins whose response to SA treatment had a significant probability value (*p* ≤0.05) across all three replicates. This ensured that any changes arising from random errors within any individual replicate experiment were excluded. Finally, a comparison between a protein's response to SA and to ATP+SA was made by performing a Student's *t* test on the averages of SA/Control and “ATP+SA”/Control ratios. All proteins with a significant probability value (*p* ≤0.05) had their response to SA attenuated by inclusion of ATP. These constitute the final protein list of this study.

##### Bioinformatic Analysis

Peptide sequences of all the proteins were analyzed using the SignalP 4.0 tool (28), which identifies the presence of an N-terminal signal peptide targeting the protein to the secretory pathway. AgriGo version 1.2 (29) was used for Gene Ontology and enrichment analysis. A total of 33 SA- and ATP-responsive proteins were submitted for enrichment analysis against an *Arabidopsis* reference database (TAIR9) with 37,767. An FDR-adjusted *p* value <0.05 was used as a cut-off threshold for a significant enrichment.

##### Confirmatory 2D-DiGE Analysis

Analysis of protein samples using 2D-DiGE was performed as previously described (7), with minor modifications. Four replicates of Control, SA, and ATP+SA protein samples were labeled with a two-dye system, in which each sample was labeled with Cye-5 and the pooled standard labeled with Cye-3. Using previous information regarding protein spot identity of *Arabidopsis* secreted proteins, CYCLASE1 protein spots were targeted for quantitative analysis. These same protein spots were excised from preparative gels and identified by tandem-MS as previously described (7).

## RESULTS

### 

#### 

##### Experimental System for Cell Death-Regulatory Protein Discovery

**Fig. 1.**
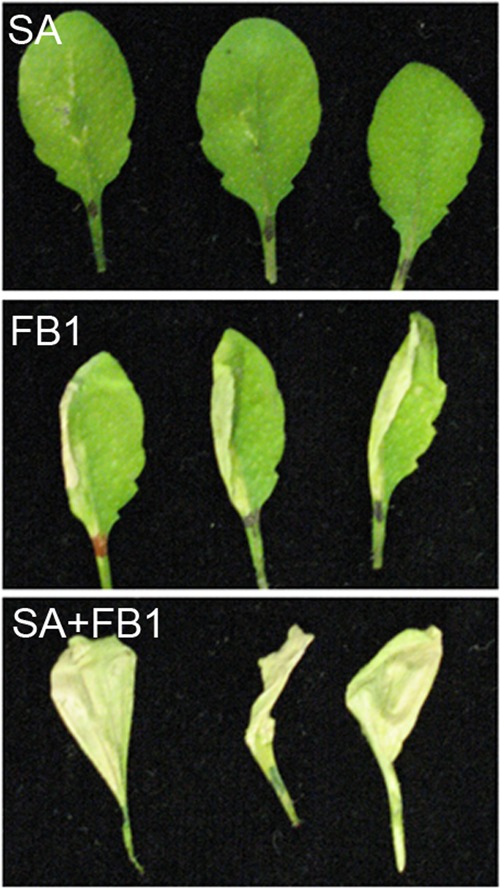
**Salicylic acid promotes FB1-induced cell death.** The left half of *Arabidopsis* leaves was infiltrated with solutions of SA (*top* panel), FB1 (*middle* panel), or SA mixed with FB1 (*bottom* panel). The leaves were detached from plants for photographing 4 days after treatment.

We reasoned that an agonist and antagonist of FB1-induced cell death could be used to design an experimental system to identify plant pcd regulatory proteins. Exogenous ATP, our chosen antagonist, blocks FB1-induced cell death in *Arabidopsis*. A previous study (17) reported that SA-deficient transgenic *Arabidopsis* plants expressing a bacterial SA-degrading enzyme are resistant to FB1, suggesting that exogenous SA might have an agonistic effect on FB1-induced pcd. We confirmed the ability of exogenous SA to promote FB1-induced death by treating one half of *Arabidopsis* leaves with solutions of SA, FB1, or a mixture of FB1+SA. Infiltration of FB1 resulted in the death of the directly treated tissues, whereas spiking the FB1 solution with SA activated an aggressive cell death that rapidly spread from the directly treated half and engulfed the whole leaf and petiole (Fig. 1). Application of a control solution of SA on its own did not affect viability of the *Arabidopsis* leaves (Fig. 1). This shows that exogenous SA promotes FB1-induced cell death in *Arabidopsis*. We hypothesized that treatment of *Arabidopsis* cell cultures with exogenous ATP and SA, in the absence of the FB1 cell death trigger, should reveal primed pcd regulatory proteins. As these two signal regulators are antagonistic, ATP should be able to block SA-mediated changes in the abundance of proteins that control cell death. Thus, proteins whose response to SA is blocked or attenuated by ATP are putative pcd regulatory candidates.

##### iTRAQ Analysis

To identify novel proteins with a putative pcd regulatory function, we used high throughput quantitative proteomic analyses of extracellular matrix proteins derived from *Arabidopsis* cell suspension cultures. Peptides arising from the samples were labeled with isobaric tags and analyzed by LC-MS/MS. To account for biological variation and ensure only reproducible responses to treatments were selected, three independent biological replicate experiments were performed. The three experiments gave a combined total of 192 proteins positively identified using the target database. Randomizing the database and performing searches with the same parameters gave no single protein identification across the three experiments, giving an Aggregate False Discovery Rate of 0. This provides greater confidence in the data sets obtained from the experiments. Peptides with sequences failing to discriminate between closely related proteins were excluded, leaving 185 nonredundant protein identifications. Of these 185 proteins, 141 were present across all three replicate experiments and so were used for subsequent analyses. The two treatments, SA and ATP+SA, were compared with the control and a fold-change ratio (relative to the control) generated for each individual protein. The ratios were averaged across the three replicates and a standard error of the mean calculated. Additional data relating to protein identification and descriptive statistics for the quantification are presented in supplemental Tables S1 and S2 and supplemental Mass Spectra.

SA treatment activated the differential expression of 78 out of the 141 proteins (Confidence ≥ 95%). ATP blocked or attenuated the SA response of 33 of these proteins (Table I). That ATP does not block all SA-induced changes in the proteome indicates the level of specificity in its antagonistic effects on SA-dependent signaling pathways. These 33 proteins provide a candidate list of putative cell death regulatory proteins.

**Table I:** List of SA-responsive proteins whose response to SA was attenuated by exogenous ATP

Gene Identifier*^a^*	Protein name	SA/Control		ATP+SA/Control		SA/ATP+SA		Signal Peptide*^f^*
		Ratio*^b^*	*p* value*^c^*	Ratio*^b^*	*p* value*^c^*	Ratio*^d^*	*p* value*^e^*	
**Proteases**								
At5g67360	Cucumisin-like serine protease (ARA12)	1.11	3.7e-4	−1.04	0.97	1.15	1.5e-2	+
At1g32960	Subtilase family protein SBT3.3	2.08	1.9e-21	1.62	3.6e-13	1.28	2.4e-2	+
At5g19120	Aspartyl protease family protein	1.77	1.3e-6	1.10	2.8e-5	1.62	3.4e-2	+
At2g05920	Subtilase family protein	−1.91	2.7e-29	−1.54	1.3e-19	−1.24	1.0e-2	+
At3g54400	Aspartyl protease family protein	−2.52	1.1e-26	−1.75	6.2e-30	−1.44	9.7e-3	+
At3g61820	Aspartyl protease family protein	−1.34	6.3e-3	1.17	0.28	−1.57	6.0e-3	+
**Oxidoreductases**								
At4g20830	FAD-binding Berberine family protein	−1.17	1.8e-4	−1.04	0.70	−1.13	4.4e-2	+
At5g05340	Peroxidase 52	1.43	0	1.09	2.3e-2	1.31	2.8e-2	+
At5g19880	Peroxidase superfamily protein	2.78	9.7e-3	1.68	3.5e-2	1.66	3.5e-3	+
At5g21105	l−Ascorbate oxidase	−1.56	4.4e-37	−1.20	2.8e-9	−1.30	1.5e-2	+
At5g44390	FAD-binding Berberine family protein	−1.68	1.3e-14	−1.16	1.4e-3	−1.45	1.8e-3	+
At5g64120	Peroxidase 71	1.55	0	−1.02	8.7e-2	1.58	1.6e-2	+
At5g51480	SKU5 similar 2	1.37	6.7e-3	1.16	0.06	1.18	4.4e-2	+
**Glycosyl−hydrolases/Glycosidases**								
At1g55120	β-Fructofuranosidase 5	1.83	1.8e-7	1.31	3.9e-4	1.40	5.5e-4	+
At1g68560	α-Xylosidase 1	−1.89	0	−1.28	2.9e-17	−1.48	7.7e-4	+
At5g63810	β-Galactosidase 10	−1.60	1.1e-4	−1.18	4.7e-2	−1.35	8.5e-3	+
At5g42720	Glycosyl hydrolase family 17 protein	2.04	4.0e-5	1.60	1.2e-3	1.28	4.1e-2	+
At2g44450	β-Glucosidase 15	−1.41	6.6e-25	−1.13	1.2e-5	−1.24	9.5e-3	+
At4g34480	O-Glycosyl hydrolases family 17 protein	1.87	0	1.59	8.5e-34	1.18	4.8e-2	+
At3g14920	Peptide-N4-(N-acetyl-beta-glucosaminyl) asparagine amidase A protein	−1.69	5.4e-5	−1.26	1.7e-3	−1.27	4.6e-3	+
At4g34260	Altered xyloglucan 8	−1.70	4.9e-11	−1.40	9.2e-9	−1.22	4.8e-3	+
At4g30270	MERI 5 protein	2.24	1.1e-2	1.31	0.24	1.70	4.0e-2	+
**Unclassified**								
At5g06870	Polygalacturonase inhibiting protein 2	−1.94	0	−1.38	1.9e-32	−1.41	3.4e-3	+
At3g54420	Class IV chitinase	1.92	5.0e-6	1.58	5.2e-5	1.21	4.0e-2	+
At1g49740	PLC-like phosphodiesterase family	−1.88	2.8e-3	−1.43	9.3e-3	−1.31	3.1e-2	+
At4g26690	Glycerophosphodiester phosphodiesterase–like 3	1.17	3.8e-2	1.00	0.84	1.16	2.7e-2	+
At4g34180	Cyclase family protein	2.01	5.0e-27	1.35	7.8e-19	1.49	1.4e-2	+
At3g15356	Legume lectin family protein	−1.57	2.8e-10	−1.04	8.8e-2	−1.51	7.1e-3	+
At1g18980	Expressed protein	2.12	3.5e-3	1.57	1.0e-3	1.35	2.7e-2	+
At5g14450	GDSL-like lipase	−1.54	4.3e-19	−1.08	0.29	−1.42	5.2e-3	+
At1g33590	Leucine-rich repeat family protein	−1.18	4.3e-6	1.11	7.5e-3	−1.31	1.1e-3	+
At5g45280	Pectinacetylesterase family protein	−1.75	1.0e-19	−1.30	1.5e-10	−1.35	1.5e-2	+
At5g55480	Glycerophosphodiester phosphodiesterase–like 4	1.22	5.7e-3	1.03	0.58	1.19	1.2e-2	+

*^a^* Arabidopsis gene identifier as annotated in the TAIR database (http://www.arabidopsis.org).

*^b^* Ratio represents the average fold-change (*n* = 3) induced by treatment relative to the control. Negative values indicate a down-regulation.

*^c^* Probability value of the quantitative difference between the treatment and control protein abundance being due to chance alone. The highest p value among the three replicate experiments is displayed. Full data set is in supplemental Table 1.

*^d^* Ratio of average protein fold-change in response SA and ATP+SA treatments (n = 3). This indicates the level by which ATP attenuated a protein's response to SA treatment.

*^e^* Probability value arising from a Student's t-test comparing the average fold-change of SA treatments to the average fold-change of ATP+SA treatments (n = 3).

*^f^* A positive sign denotes the presence of a predicted signal peptide in the primary sequence of the protein.

##### Differentially Expressed Proteins Attenuated by ATP

**Fig. 2.**
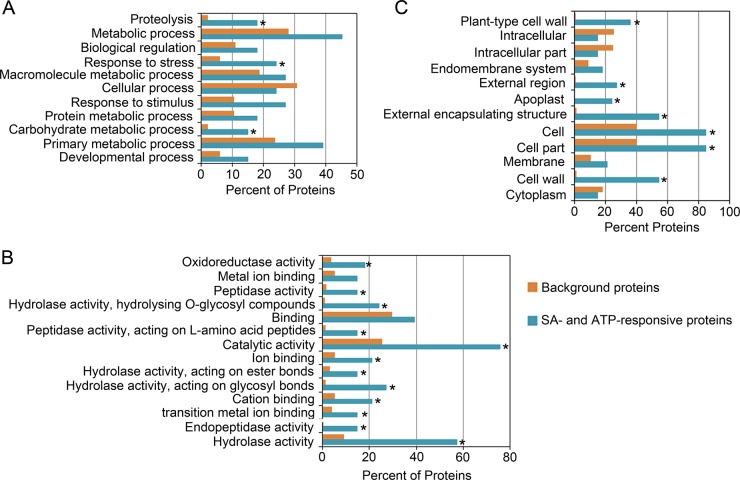
**Gene Ontology analysis of SA- and ATP-responsive proteins.** *A*, Biological process. *B*, Molecular function. *C*, Cellular component. Significantly overexpressed terms (FDR-adjusted *p* value < 0.05) relative to the background proteins (TAIR9 database) are marked with an asterisk.

A total of 33 proteins were identified as putative cell death regulatory proteins on the basis of their response profile to SA and ATP treatments (Table I). ATP attenuated the exogenous SA-triggered differential expression of these proteins. All 33 proteins were submitted for GO annotation and enrichment analysis against a background reference dataset of the GO annotation of the total *Arabidopsis* genome. Fig. 2 shows the distribution charts of biological process, molecular function, and cellular component. A very broad range of molecular functions was significantly enriched in this data set, and it included oxidoreductase activity, hydrolase activity, peptidase activity, and ion binding (Fig. 2*B*). Proteins with these molecular functions were annotated as components of carbohydrate metabolic processes, proteolysis, and response to stress, which are the main significantly enriched biological processes (Fig. 2*A*). However, there was no clear pattern or trend in respect of the direction of the response within any of the three protein groups. Thus, each group had some proteins up-regulated and some down-regulated by SA treatment (Table I). In accordance with extracellular matrix localization, all the 33 proteins possess a predicted N-terminal signal peptide (Table I), which targets the polypeptide to the endoplasmic reticulum (30), and no endoplasmic reticulum-retention signal (31). An obvious enrichment for extracellular matrix proteins was confirmed by GO enrichment analysis (Fig. 2*C*). However, it appears that some of these proteins may also exist in other intracellular compartments (Fig. 2*C*), raising the possibility that some of these proteins may relocate between compartments in response to internal and external cues. Alternatively, as all extracellular proteins transit through the endoplasmic reticulum and Golgi complex, they can be captured in the endomembrane system while en route to the extracellular matrix. These proteins are specifically secreted to the extracellular matrix, as there was no evidence of cell lysis based on the absence of known cytoplasmic proteins in the protein preparations. The proteins identified here (Table I) are predicted to be extracellular using SignalP and most of them have been independently identified in the *Arabidopsis* extracellular matrix in previous proteomic studies, such as the 2008 study of Kaffarnik *et al.* (32).

##### CYCLASE1 Regulates Plant PCD

After identification of putative death regulatory proteins, the next stage was to provide definitive evidence for their function in cell death. This aspect of the research will inevitably take a long time to complete, but in this study we validate the approach using a selected single candidate protein. The candidate protein was selected from the list using the following screening and logic. A short-list of seven proteins was selected from Table I based on a threshold of twofold response to SA treatment. Next, the extent of suppression of the SA response by ATP (SA/ATP+SA ratio; Table I) was used to rank the seven proteins and the top three candidates were MERI 5 protein (At4g30270), peroxidase superfamily protein (At5g19880), and cyclase family protein (At4g34180), respectively. The goal was to select a candidate from a small gene family to enhance the chances of obtaining a biologically relevant phenotype in T-DNA gene knockout mutants because of the relatively diminished probability of gene redundancy when compared with big gene families. MERI 5 is a xyloglucan endotransglucosylaase/hydrolase-24 belonging to a 33-member gene family in *Arabidopsis* (33), whereas there are 73 expressed genes in the *Arabidopsis* peroxidase multigene family (34). As a result, we selected the cyclase family protein (At4g34180) for further analysis.

**Fig. 3.**
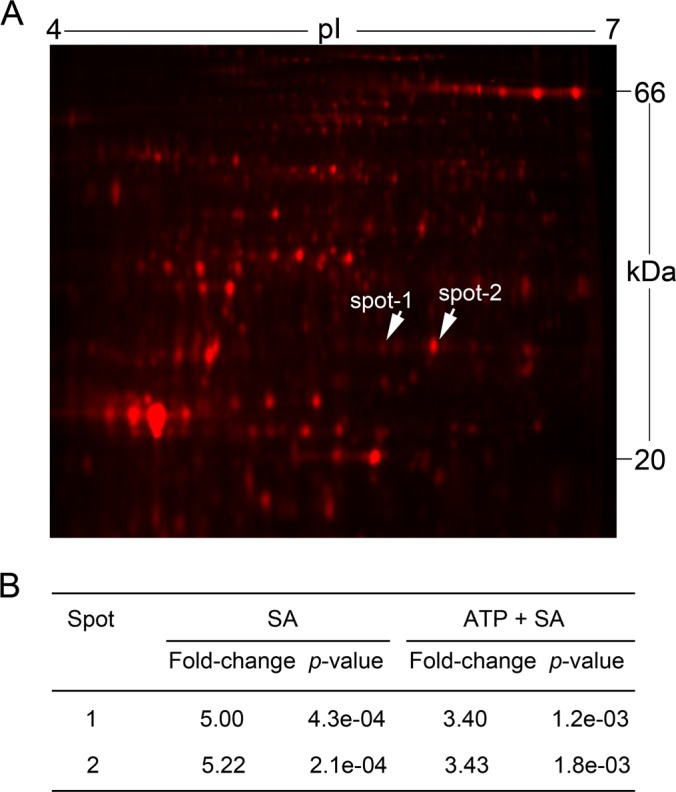
**Gel-based analysis of CYCLASE1 responses.** *A*, 2-dimensional gel of proteins secreted into the growth medium of *Arabidopsis* cell suspension cultures. CYCLASE1 protein was identified in spot-1 and spot-2 indicated by arrows. *B*, CYCLASE1 protein spot abundance analyzed by 2D-DiGE in samples treated with SA or ATP+SA.

At4g34180 is annotated in the database as a cyclase family protein because of possession of a putative cyclase domain, which is found in antibiotic synthetic enzymes (35). However, neither cyclase enzymatic activity nor any physiological function for this protein has been reported. We named this protein CYCLASE1 because the *Arabidopsis* genome has two additional closely related genes coding for secreted proteins with the same cyclase domain. Accordingly, we named the related genes *CYCLASE2* (At4g35220) and *CYCLASE3* (At1g44542). The twofold increase in CYCLASE1 protein in response to SA was suppressed by ATP down to 1.35-fold (Table I). On 2-dimensional gels, CYCLASE1 was identified in two protein spots of the same molecular weight but different isoelectric points (Fig. 3*A*). Analysis by 2-dimensional difference gel electrophoresis revealed that both CYCLASE1 protein spots are up-regulated ∼fivefold by SA and ATP attenuates this down to 3.4-fold (Fig. 3*B*). There is a noticeable difference in the SA-induced CYCLASE1 fold-change obtained via iTRAQ (Table I) and the value from 2D-DiGE (Fig. 3*B*). This discrepancy may arise for to two reasons. First, most proteins appear in protein gels as several protein spots, because of differential post-translational modifications, with the possibility that a fixed pH range of the first dimension gel might not accommodate the isoelectric points of all the charge variants of the protein. Thus, there could be other spots of CYCLASE1 protein outside of the pH 4–7 range used in this study, which were missed by 2D-DiGE analysis and could have possibly brought the average fold-change down to ∼twofold. Second, post-translational modifications such as proteolytic cleavage, can shift the position of the proteolytic products in gels that the only way to identify these is to pick the entire gel and identify every single protein spot. Thus, other spots of CYCLASE1 might have been missed from the 2D-DiGE analysis this way. In contrast, iTRAQ is performed in solution and so all the CYCLASE1 peptides, irrespective of post-translational modification, will be identified and accounted for in the quantitative analysis. Notwithstanding the observed discrepancy, both iTRAQ and 2D-DiGE revealed that ATP attenuates the SA-induced increase in CYCLASE1.

**Fig. 4.**
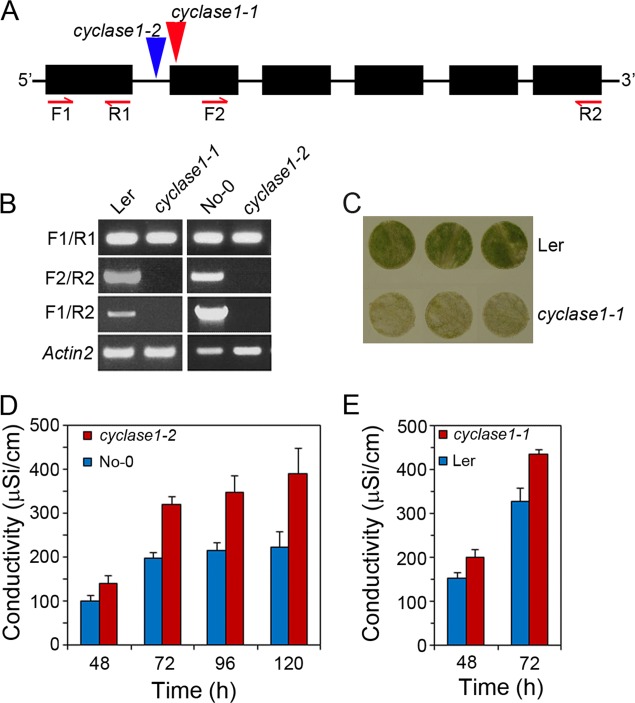
***CYCLASE1* gene knockout predisposes mutants to an accelerated cell death phenotype.** *A*, Schematic digram showing the *CYCLASE1* gene structure with six exons (black rectangles) and the relative positions of T-DNA insertions (inverted triangles) in the mutants. *B*, RT-PCR amplification of *CYCLASE1* fragments in cDNA samples of wild type (No-0 and Ler) plants and T-DNA knockout lines *cyclase1–1 cyclase1–2* using the reverse (R) and forward (F) primers indicated in panel *A. Actin-2* was used as a constitutive reference control. *C*, Appearance of wild type Landsberg *erecta* (Ler) and *cyclase1–1* leaf discs floating on FB1 for 3 days. *D*, The conductivity of solutions on which FB1-treated leaf discs floated was plotted against time to give the cell death kinetic profile of wild type Nossen-0 (No-0) and *cyclase1–2* plants. *E*, Cell death profiles of FB1-treated leaf discs from wild type (Ler) and *cyclase1–1* plants. Values and error bars represent means ± S.D. (*n* = 5). The difference between wild type and mutant plants is statistically significant (*p* ≤ 0.05) across all time-points.

To investigate the role of CYCLASE1 in pcd, two independent T-DNA insertion mutants were isolated from JIC SIM (22) and RIKEN (23, 24) collections. *CYCLASE1* gene has six exons, and the chosen T-DNA mutants have insertions in exon-2 and in the 5′ intron of exon-2 (Fig. 4*A*). In homozygous lines GT_5_42439 (*cyclase1–1*) and RATM13_3839_1 (*cyclase1–2*), which are in Landsberg *erecta* and Nossen-0 ecotypes, respectively, no *CYCLASE1*-specific transcript could be detected using primers downstream of the insertion positions (Fig. 4*A*, 4*B*). Although the exon-1 transcript, which is up-stream of the insertion positions, was expressed, primers straddling the insertion positions and covering the full coding sequence did not amplify any product (Fig. 4*A*, 4*B*). This confirmed that *CYCLASE1* gene expression was effectively disabled in the mutant lines. Next we used plants from these gene knockout lines to investigate the role of CYCLASE1 in FB1-induced cell death. We used both quantitative and qualitative assays for cell death in leaf tissues exposed to FB1. The quantitative assay relies on loading leaf discs with FB1 during a 48 h period of continuous dark incubation and then transferring the tissues to a light-dark cycle to activate cell death. As cells die, they release ions into the solution on which the discs are floating, and the conductivity of the solution increases in proportion to the level of cell death. A plot of the conductivity against time provides a cell death kinetic profile that can be used to compare between different genotypes. Both the *cyclase1–1* and *cyclase1–2* knockout lines had a statistically significant higher extent and rate of cell death than the respective wild type plant tissues (Fig. 4*C*, 4*D*). A photograph of Landsberg *erecta* and *cyclase1–1* leaf discs exposed to FB1 treatment revealed the hyper-susceptibility to FB1-induced pcd of plants lacking a functional gene of CYCLASE1 (Fig. 4*B*). We also noted ecotype differences in pcd rate. It took the Landsberg *erecta* knockout plants (*cyclase1–1*) only 72 h to breach a conductivity value of 400 μSi/cm, whereas *cyclase1–2*, which is in the Nossen-0 ecotype, reached this level of pcd at ∼120 h (Fig. 4*D*, 4*C*, respectively). We terminate the conductivity measurements when the value reaches 450–500 μSi/cm as the assay ceases to be linear beyond this point. Overall, these results show very clearly that CYCLASE1 is a negative regulator of FB1-induced pcd.

##### Response of CYCLASE1 T-DNA Knockout Mutants to Pathogens

**Fig. 5.**
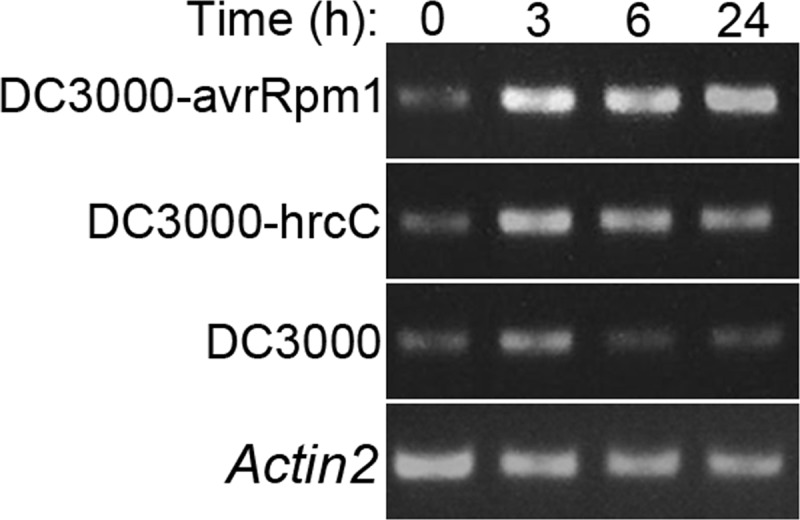
**Response of *CYCLASE1* gene expression to pathogens.** *Arabidopsis* plants were inoculated with the indicated mutant strains of *Pseudomonas syringangae* pv. *tomato* DC3000 and tissues for RNA extraction harvested at the indicated time-points. RNA samples were analyzed by RT-PCR amplification of *CYCLASE1*. A representative gel of the constitutive reference control gene *Actin-2* is provided in the bottom panel.

As pcd is part of the hypersensitive response to pathogen attack, we wondered if CYCLASE1 might be involved in this defensive response. First, we monitored *CYCLASE1* gene expression in leaf tissues inoculated with two types of bacterial pathogen. Strain DC3000 of the bacterial pathogen *Pseudomonas syringae* pv. *tomato* causes bacterial speck disease in *Arabidopsis*. However, a strain of DC3000 carrying a plasmid expressing the avrRpm1 protein is intercepted by the *Arabidopsis* pathogen surveillance system, which activates the defensive hypersensitive response characterized by a rapid pcd of cells in the vicinity of the infection and synthesis of antimicrobial proteins and secondary metabolites in the entire leaf. *CYCLASE1* gene expression was up-regulated within 3 h of inoculation with DC3000-avrRpm1, with the elevated transcripts being maintained to 24 h postinoculation (Fig. 5). However, the plants similarly activated *CYCLASE1* transcript accumulation in 3 h of exposure to the virulent strain DC3000, but this was rapidly suppressed by 6 h and remained low through to 24 h (Fig. 5). The differential response to these pathogens suggested that CYCLASE1 might possibly influence pathogen-induced pcd and development of disease.

**Fig. 6.**
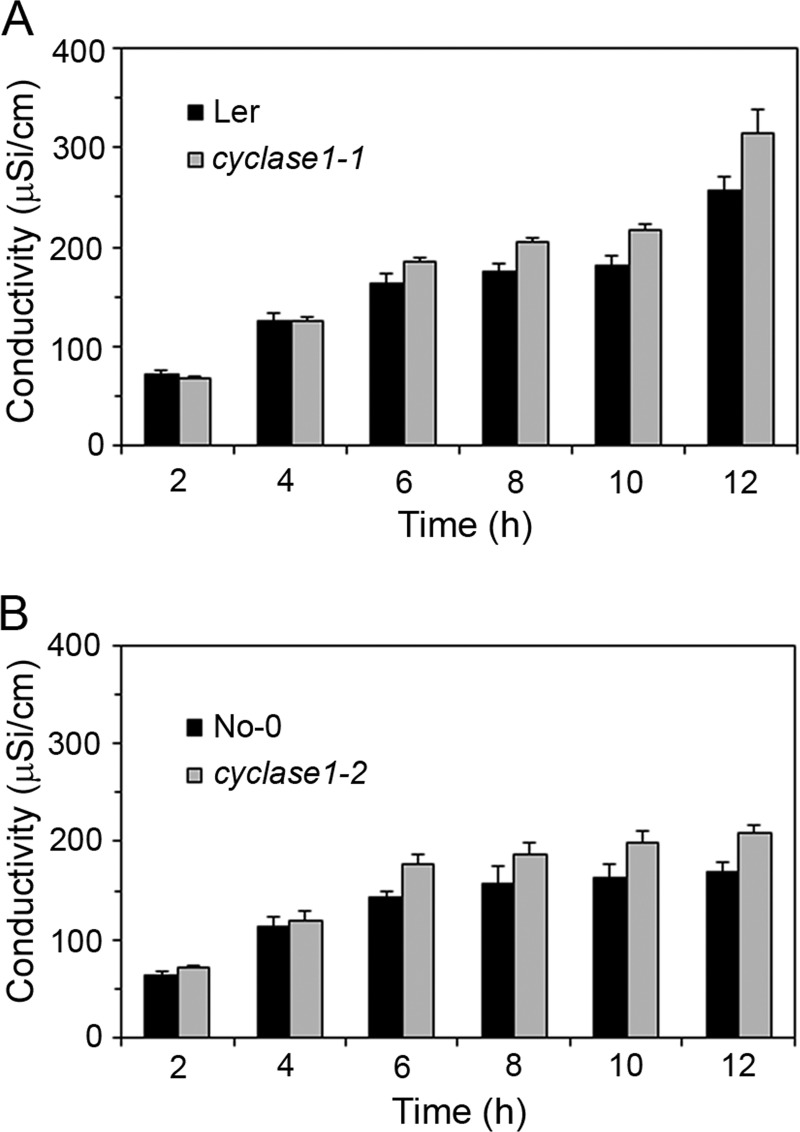
***CYCLASE1* gene knockout increases hypersensitive pcd.** Discs were cored from *Arabidopsis* leaves inoculated with DC3000-avrRpm1 and floated on water for conductivity measurements. *A*, Cell death profiles of Landsberg *erecta* (Ler) and *cyclase1–1. B*, Cell death profiles of Nossen-0 (No-0) and *cyclase1–2*. Values and error bars represent means ± S.D. (*n* = 5). The difference between wild type and *CYCLASE1* knockout mutants is statistically significant (*p* ≤ 0.05) from 6–12 h.

**Fig. 7.**
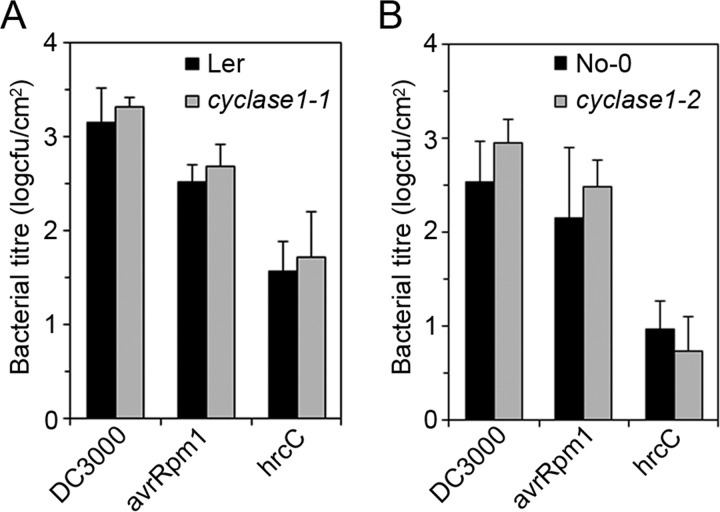
**Absence of CYCLASE1 does not alter pathogen multiplication.** Plants were inoculated with DC3000, DC3000-avrRpm1 (avrRpm1), or DC3000-hrcC (hrcC). Bacterial titer in the tissues was enumerated 3 days or 6 days postinoculation for DC3000/DC3000-avrRpm1 and DC3000-hrcC, respectively. Bacterial colony forming units (cfu) expressed as log_10_. *A*, Comparison of bacterial titer between Landsberg *erecta* (Ler) and *cyclase1–1. B*, Comparison of bacterial titer between Nossen-0 (No-0) and *cyclase1–2*. Values and error bars represent means ± S.D. (*n* = 3). The difference between wild type and *CYCLASE1* knockout mutants across all pathogens is not statistically significant (*p* > 0.05).

**Fig. 8.**
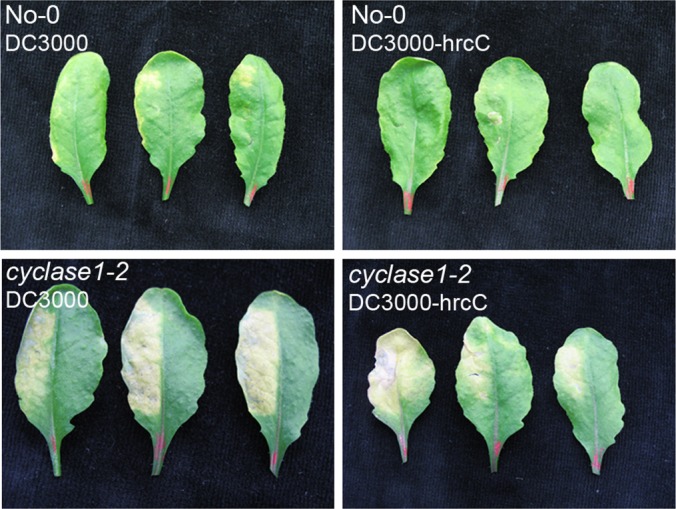
***CYCLASE1* knockout mutants develop severe disease symptoms.** *Arabidopsis* leaves were inoculated with either DC3000 or DC3000-hrcC. Leaves were detached from the plants 3 days (DC3000) or 6 days (DC3000-hrcC) postinoculation for photography. The marks on the middle No-0 leaf inoculated with DC3000-hrcC are pressure-induced wounding marks because of the inoculation process, otherwise these leaves develop no symptoms at all. Note; only the left half of each leaf was inoculated.

We used the conductivity assay to compare the cell death kinetic profiles of wild type plants and *CYCLASE1* gene knockout mutants. Leaf discs cored from tissues infiltrated with an inoculum of DC3000-avrRpm1 were floated on water for conductivity measurements. Both the *cyclase1–1* and *cyclase1–2* knockout plants responded to DC3000-avrRpm1 with a higher and more rapid cell death than the wild type (Fig. 6). In contrast to the big increase in FB1-induced pcd (Fig. 4), the increase in pathogen-induced pcd in *CYCLASE1* gene knockout mutants was relatively lower, though still statistically significant between 4–12 h (Fig. 6). Taken together, the response of *CYCLASE1* gene expression to pathogens and the increased hypersensitive cell death in gene knockout mutants raised the possibility for a role of CYCLASE1 in plant-pathogen interactions. Thus, we monitored the multiplication of bacterial pathogens in inoculated *Arabidopsis* tissues. There were no statistically significant differences in pathogen multiplication between wild type and knockout mutant plants after inoculation with either the virulent DC3000 or the avirulent DC3000-avrRpm1 (Fig. 7). This indicates that CYCLASE1 does not affect pathogen multiplication. However, an unexpected observation we made in the course of these experiments was the appearance of extreme chlorosis symptoms in the knockout plants inoculated with the virulent pathogen DC3000 (Fig. 8). Bacterial disease symptoms are dependent on the ecotype, age of plants, temperature, and light. Under our experimental conditions, wild type Nossen-0 plants infected with virulent DC3000 exhibit very mild patchy chlorosis (Fig. 8). The dramatic disease symptoms in the *cyclase1* knockout plants were not a result of increased bacterial multiplication (Fig. 7), but rather an altered tissue reaction to colonization by the bacteria.

To further explore this phenomenon, we used another strain of DC3000 that has a disabled *hrcC* gene. The virulent strain DC3000 delivers virulence factors into host plant cells via the type-3 secretory system in order to suppress host defenses and establish a successful infection. The mutation in DC3000-hrcC disrupts the type-3 secretory system, rendering the mutant a weak pathogen that hardly grows *in planta* and certainly fails to evoke any disease symptoms. In wild type plants DC3000-hrcC also activates *CYCLASE1* gene expression in a similar fashion to DC3000-avrRpm1 (Fig. 5). *CYCLASE1* gene knockout plants inoculated with DC3000-hrcC developed the same striking symptoms similar to those evoked by the virulent strain in the *cyclase1* knockout plants (Fig. 8). Moreover, determination of bacterial titer in these plants revealed no differences between the wild type and *CYCLASE1* gene knockout plants inoculated with DC3000-hrcC (Fig. 7). Thus, a pathogen that fails to multiply in *Arabidopsis* is capable of triggering remarkable chlorosis in the knockout plants. Overall, our study shows that CYCLASE1 is a negative regulator of pcd as well as bacterial disease symptoms in *Arabidopsis*. This shows the power of proteomics as a discovery tool in biology.

## DISCUSSION

### 

#### 

##### Targeting the Extracellular Matrix to Identify PCD regulatory Proteins

We set up an experimental system in which we targeted soluble proteins in the extracellular matrix, which are responsive to a pcd agonist and antagonist. The advantage of the experimental system is that it uses SA and ATP, treatments that do not cause cell death and so avoids death-induced secondary effects on the proteome. In addition, restricting the proteomic analyses to the mobile phase of the extracellular matrix provides a unique opportunity for new functional protein discoveries. This is particularly important because the extracellular compartment has little been researched with a view that it is a central hub coordinating cellular responses at tissue level. There is growing evidence showing that extracellular matrix signals connect to and control nearly all aspects of plant growth and development, as exemplified by brassinosteroid. Brassinosteroid binds to the extracellular domain of its receptor kinase, brassinosteroid insensitive 1 (BRI1), to activate cytoplasmic signaling and gene expression affecting other signaling pathways such as auxin, light, and gibberellin pathways (36). Cell–cell signaling via the extracellular matrix should inevitably involve transmission of mobile signals through the apoplastic fluid. The signals include small metabolites, peptides, protein ligands, and signal regulatory proteins. In this study, we focused on the protein fraction of the apoplastic fluid equivalent of cell suspension cultures. A total of less than 200 proteins were identified, indicating how simple the proteome is in comparison with total protein extracts, which can have thousands of proteins (37). A staggering 55.3% of the identified proteins were responsive to SA treatment, supporting the hypothesis that perception of a signal can trigger a resetting of cell–cell communication networks reflected by a quantitative shift in the mobile phase proteome. The large number of responsive proteins may reflect an upsurge in signal initiation, propagation, or termination as cells reset their metabolism and communicate this to their neighbors. Use of this system has now enabled us to identify an important regulator of pcd and bacterial disease symptoms.

##### Putative Cell Death Regulatory Proteins

A number of peroxidases and other oxidoreductase enzymes were among the proteins identified as putative pcd regulators. Although peroxidases have established roles in lignification (38) and cross-linking of structural cell wall proteins (39), a direct role in cell death signaling also exists. Reactive oxygen species are an important pcd-associated signaling component produced during FB1-induced (15) and pathogen-induced (40) cell death. Extracellular peroxidases and plasma membrane-bound NADPH-oxidases are major sources of reactive oxygen species in pathogen defense and pcd signaling. Silencing an extracellular peroxidase of *Capsicum annuum* abolished H_2_O_2_ accumulation and hypersensitive response pcd (41). *Arabidopsis* mutants lacking the membrane-bound AtrbohD and AtrbohF oxidases generate no reactive oxygen species and have reduced hypersensitive cell death in response to pathogen infection (42). Thus, the extracellular oxidoreductase proteins identified in this study could function by modulating the levels of apoplastic reactive oxygen species to control propagation and termination of pcd across cells within a given tissue. The fact that these enzymes respond to the pcd agonist and antagonist raises the possibility that the effects of SA and ATP on FB1-induced pcd could be mediated via this group of proteins.

The largest group of proteins that responded to SA and ATP treatments are glycosyl hydrolases, which modify cell walls by degrading polysaccharides such as pectins, arabinogalatoproteins, and xyloglucans. Although it is not clear how this might affect pcd, we can only speculate based on previous reports how this might impinge on cell death. First, the integrity of the cell wall-plasma membrane connections is important to maintain cell viability, because destabilizing the protein connections by Yariv reagent, a chemical that specifically binds and aggregates arabinogalactan proteins activates pcd (43). Second, sugars released by the degradation of cell wall polysaccharides might be used as potent cell signaling molecules in promoting pcd. There is evidence that sugar-mediated signaling promotes FB1-induced pcd (44). Thus, in addition to releasing signal molecules for cell–cell communication from the cell wall, glycosyl hydrolase activity has the potential to prime cells for pcd by modifying cell wall-plasma membrane structural configuration.

Extracellular proteases featured prominently in the list of proteins responsive to SA and ATP treatments (Table I). That proteases are regulators of plant pcd is not surprising, given that a variety of protease inhibitors are known to abolish cell death triggered by pathogens and pcd elicitors such as H_2_O_2_, chitosan, and xylanase (45). However, identification of a group of extracellular matrix proteases as putative pcd regulators is very significant as it implicates protease networks in cell–cell signaling during programmed cell death. A role for extracellular proteases in plant pcd is supported by the observation that exogenous trypsin activated pcd in zinnia, which was dependent on Ca^2+^ influx into the cytosol (46). In *Arabidopsis*, overexpression of an extracellular aspartic protease activates spontaneous micro-lesions of pcd (47). The proteases could function in enzyme activation by cleaving off auto-inhibitory peptides, or in production of bioactive peptides required for downstream pcd signaling.

A number of unclassified proteins with diverse biochemical activities were also identified as potential pcd regulatory proteins. We note that exogenous ATP has previously been reported to also block SA-induced changes in the abundance of pathogen defense proteins (48). It is quite possible that some of the proteins identified may have a purely defensive role without any impact on cell death. Future studies using reverse genetic approaches or transgenic plants overexpressing the target proteins should provide definitive evidence of their involvement in cell death regulation as has been done for CYCLASE1.

##### CYCLASE1 Regulates PCD

Proteomics and reverse genetics using T-DNA insertion gene knockout mutants led to the identification of CYCLASE1 as a novel extracellular matrix protein regulating pcd in *Arabidopsis*. The occasional occurrence of a phenotype in a T-DNA insertion mutant arising from a secondary mutation, which is not in the T-DNA-tagged gene indexed in the mutant collection database, can hamper this type of study. Therefore, confirmation of the observed phenotype in a second mutant line or complementation of mutant plants with the target gene is required (49–51). Therefore in this study, we used two independent T-DNA insertion gene knockout lines to confirm the role of CYCLASE1 in pcd.

Mutant plants devoid of CYCLASE1 were more susceptible to FB1- and pathogen-induced cell death, relative to wild type plants. This indicates that CYCLASE1 is a negative regulator of cell death. Interestingly, SA the prodeath hormone led to an increase in CYCLASE1 protein, which was blocked by exogenous ATP (Fig. 3*B*; Table I). This suggests that plants probably deploy CYCLASE1 in an attempt to control the “runaway” FB1-induced cell death. However, in the context of pathogen infection, the increase in CYCLASE1 (Fig. 5) becomes important to suppress the unbridled progression of chlorotic symptoms seen in pathogen-infected CYCLASE1 knockout plants (Fig. 8). This clearly explains why in wild type plants virulent DC3000 causes disease symptoms and DC3000-hrcC does not. Our data reveal that the virulent pathogen (DC3000) suppressed *CYCLASE1* gene expression in wild type plants, resulting in development of disease symptoms, whereas DC3000-hrcC does not evoke disease symptoms as the wild type plants successfully up-regulate *CYCLASE1* expression. Although disease susceptibility is usually measured by the ability of the pathogen to multiply *in planta*, our results show that damaging disease symptoms (chlorosis) can occur without a corresponding increase in bacterial titer. CYCLASE1 protein is a key regulator of this phenomenon.

Microarray data in the publicly available GENEVESTIGATOR database (52) reveal that the level of *CYCLASE1* expression is very high and remains essentially constant during development, with a slight increase at the onset of senescence (https://genevestigator.com). *CYCLASE1* is up-regulated by the defense hormone salicylic acid, but not by jasmonic acid or ethylene. Expression of *CYCLASE1* is also stimulated in response to viral, fungal, and bacterial pathogens. These include turnip mosaic virus, *Alternaria brassicicola*, and several pathovars of *Pseudomonas syringae*. Moreover, the flagellin-derived elicitor flg22 and elongation factor TU-derived elicitor elf18 massively up-regulate *CYCLASE1* expression. Thus, the response of *CYCLASE1* to pathogens, elicitors, and defense hormones suggests a broader role in *Arabidopsis* pcd and stress response.

The *Arabidopsis* genome database has three secreted putative cyclase proteins. We were able to PCR-amplify the transcripts of *CYCLASE1* and *CYCLASE2* in samples derived from *Arabidopsis* cell suspension cultures and leaf material, but failed to detect *CYCLASE3* (data not shown). This could indicate that the *CYCLASE3* gene is not expressed at all or that it might be expressed in specific organs. Attempts to generate double knockout mutants of *CYCLASE1/CYCLASE2* were unsuccessful (data not shown), probably because of a lethal phenotype. This indicates that there is gene redundancy between *CYCLASE1* and *CYCLASE2* for normal growth and development and that the protein function(s) is indispensable for viability. This function has not yet been determined biochemically. However, the function of CYCLASE1 in pcd regulation is unique, because CYCLASE2 knockout mutants do not have a similar phenotype (data not shown). This appears to rule out the cyclase domain from being important in CYCLASE1's function in pcd control. Therefore, ongoing experiments investigating the mechanism by which CYCLASE1 function are focusing on regions of CYCLASE1 protein sequence that differ from the CYCLASE2 sequence.

Although precise mechanistic details of the molecular basis for FB1-induced cell death in plants remains unclear, CYCLASE1 joins a few key regulatory proteins already identified. Gene expression regulatory proteins, signaling proteins, and metabolic enzymes are known to regulate the response of *Arabidopsis* to FB1. An SPB-domain transcription factor AtSPL14 (53) and an APETALA2/ethylene response factor (ERF) transcription factor MACD1 (54) are positive regulators of FB1-induced cell death. T-DNA insertion mutants of vacuolar processing enzyme (55), UDP-glucose pyrophosphorylase (44), ATP synthase β-subunit (7), and mitogen-activated protein kinase 6 (13) suppress FB1-triggered cell death, indicating that these proteins are FB1 antagonists. Equally, RNA interference of RING1 E3 ligase attenuates the *Arabidopsis* cell death response to FB1 (56). RNA interference lines of the *Arabidopsis* serine protease inhibitor KTI1 have enhanced FB1 cell death, whereas overexpression reduces the response (57). *Arabidopsis* homologs of animal cell death inhibitory proteins also regulate FB1-induced death. For example, T-DNA insertion mutants of At1g63900 and At1g59560, homologs of the *Drosophila i*nhibitor of *a*poptosis 1 (DIAP1) homologs, have exacerbated FB1 cell death in comparison to wild type plants (58). Similarly, mutants of the *Arabidopsis* homolog of Bax inhibitor-1 have accelerated progression of cell death after exposure to FB1 (59). Although the order in which all these proteins function is not clear, these reports indicate that multiple factors modulate the complex plant cell death response to FB1.

## Supplementary Material

Supplemental Data

## Footnotes

^1^ The abbreviations used are:

pcd: programmed cell death
2D-DiGE: 2 dimensional difference gel electrophoresis
CFU: colony forming units
DIAP: Drosophila inhibitor of apoptosis
eATP: extracellular ATP
FB1: fumonisin B1
GO: Gene Ontology
LCBs: long chain bases
Ler: Landsberg *erecta*
No-0: Nossen-0
SA: salicylic acid
T-DNA: transfer-DNA.
