# Supplementary material for: A Novel Function for Arabidopsis CYCLASE1 in Programmed Cell Death Revealed by Isobaric Tags for Relative and Absolute Quantitation (iTRAQ) Analysis of Extracellular Matrix Proteins
Source: Mol Cell Proteomics. 2015 Apr 10;14(6):1556–68. doi: 10.1074/mcp.M114.045054 (PMC4458720; doi:10.1074/mcp.M114.045054)
Supplement: Supplemental Data [file supp_14_6_1556__index.html]

A novel function for Arabidopsis CYCLASE1 in programmed cell death revealed by iTRAQ analysis of extracellular matrix proteins — A Novel Function for Arabidopsis CYCLASE1 in Programmed Cell Death Revealed by Isobaric Tags for Relative and Absolute Quantitation (iTRAQ) Analysis of Extracellular Matrix Proteins — Arabidopsis Cell Death-Regulatory Proteins — Supplemental Data 

# A Novel Function for *Arabidopsis* CYCLASE1 in Programmed Cell Death Revealed by Isobaric Tags for Relative and Absolute Quantitation (iTRAQ) Analysis of Extracellular Matrix Proteins

## Supplemental Data

**Files in this Data Supplement:**

- Supplemental Mass Spectra - Fragmentation spectra of single precursor ions used to identify and quantify proteins, for which identification was based on only 1 peptide
- Supplemental Table 1 - Details of proteins whose response to SA was attenuated by exogenous ATP
- Supplemental Table 2 - Descriptive statistics for proteins that responded to salicylic acid (SA) or ATP+SA treatments
