# Supplementary material for: A Novel Function for Arabidopsis CYCLASE1 in Programmed Cell Death Revealed by Isobaric Tags for Relative and Absolute Quantitation (iTRAQ) Analysis of Extracellular Matrix Proteins
Source: Mol Cell Proteomics. 2015 Apr 10;14(6):1556–68. doi: 10.1074/mcp.M114.045054 (PMC4458720; doi:10.1074/mcp.M114.045054)

Supplemental Mass Spectra

The fragmentation spectra of the single precursor ions (SEE Table S1) used to identify and quantify 3 proteins, for which identification was based on only 1 peptide, are given below.

At5g19880 – Peroxidase superfamily protein

Peptide sequence: GVIESDQILFSSTGAPTVSLVNR

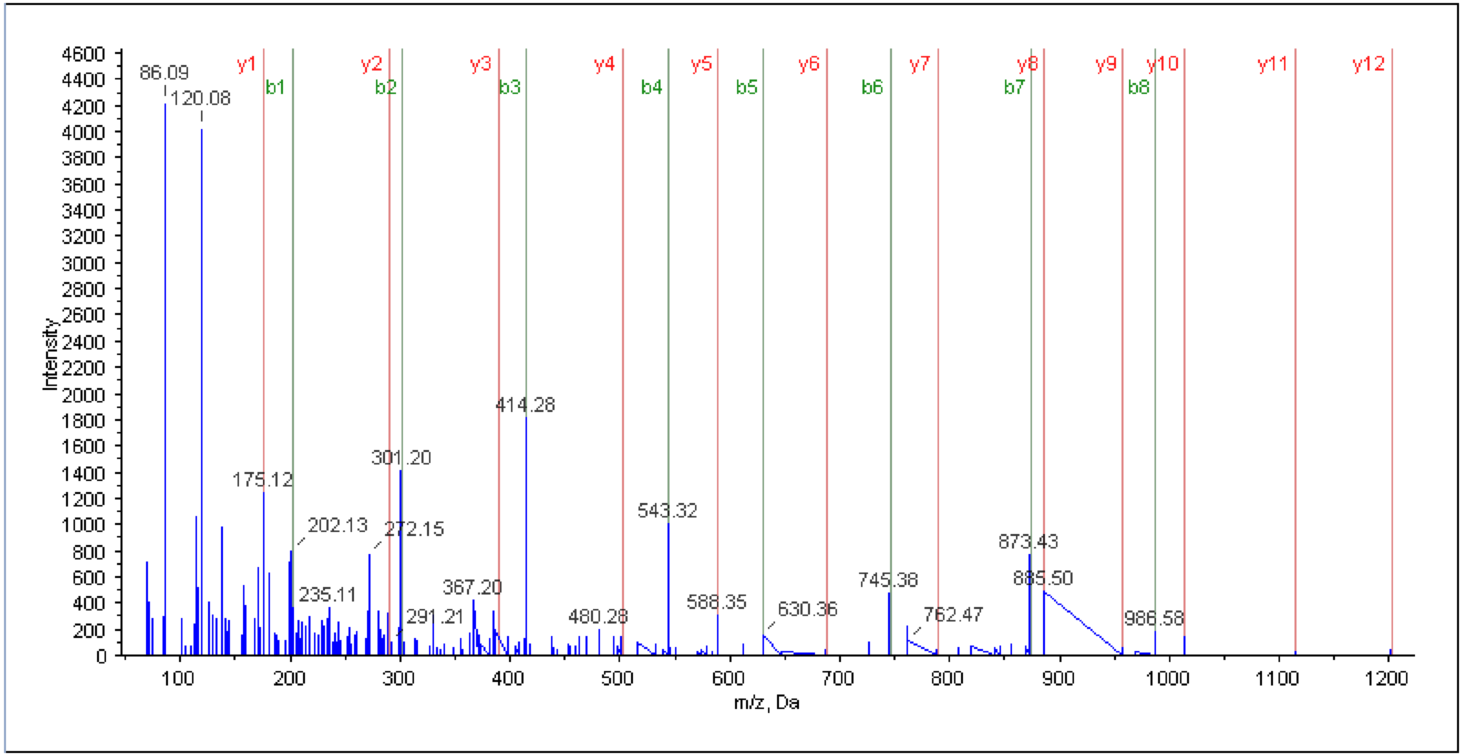

At4g30270 – MERI 5 protein
Peptide sequence: IILTVDDTPIR

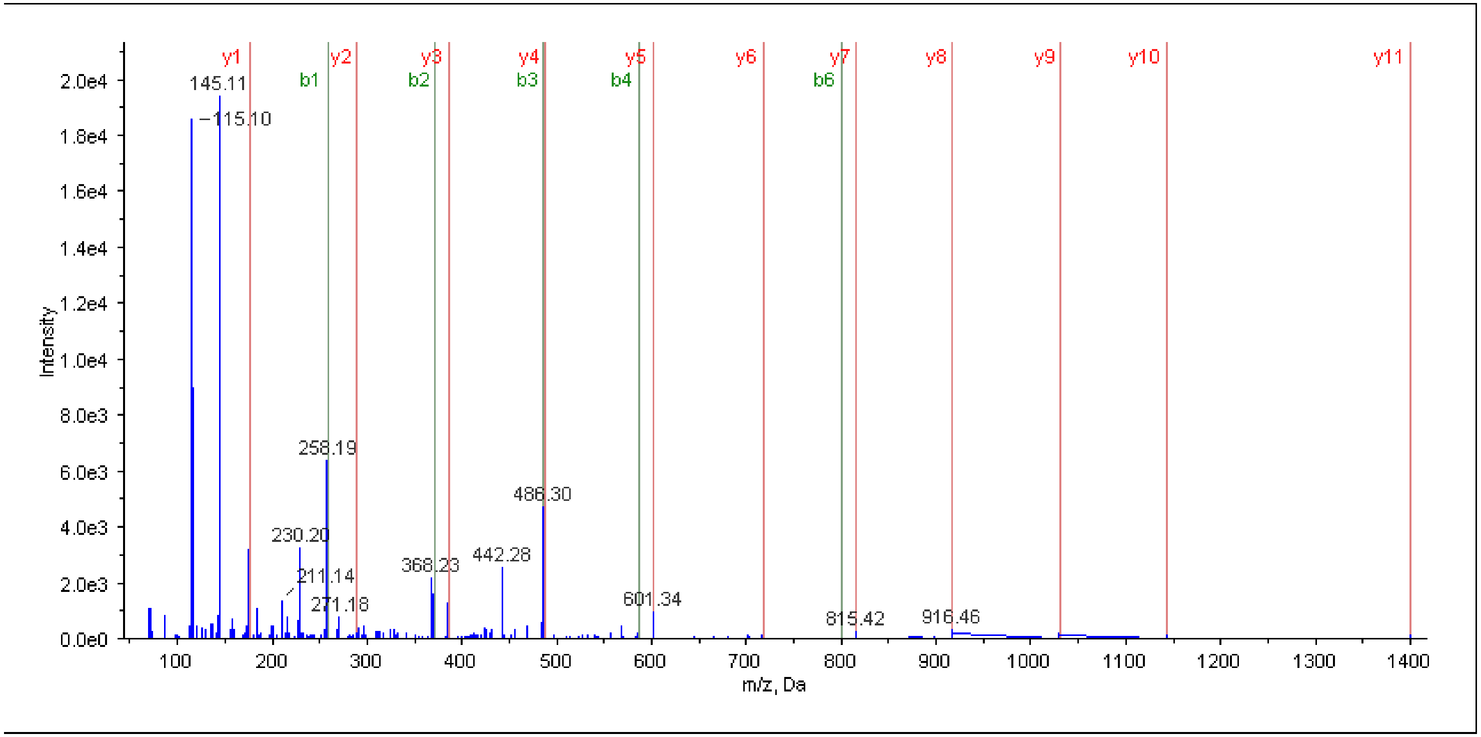

At1g18980 – Expressed protein
Peptide sequence: VLNAGEMFVVPR

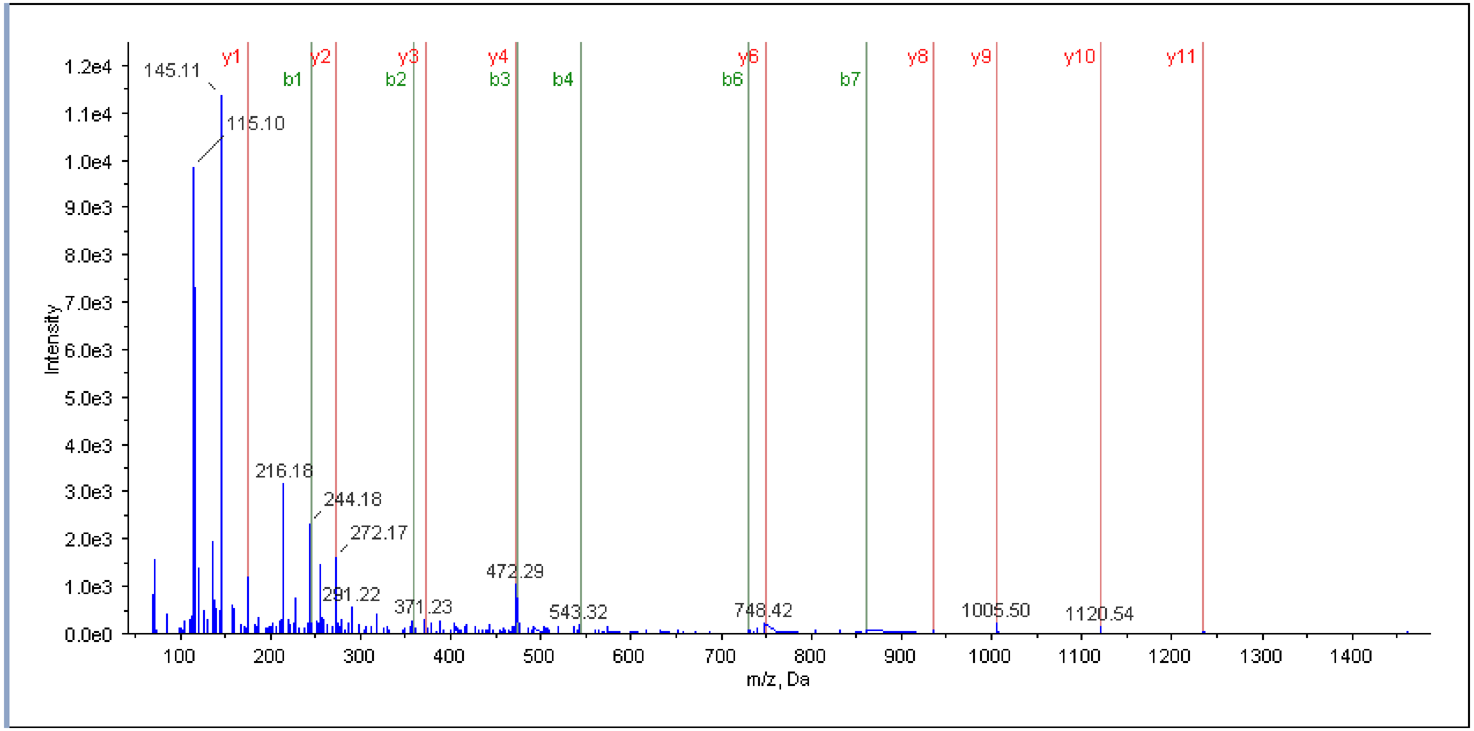

Supplement: Supplemental Data [file supp_M114.045054_mcp.M114.045054-3.pdf]
